# Cofactor-Independent Phosphoglycerate Mutase from Nematodes Has Limited Druggability, as Revealed by Two High-Throughput Screens

**DOI:** 10.1371/journal.pntd.0002628

**Published:** 2014-01-09

**Authors:** Gregory J. Crowther, Michael L. Booker, Min He, Ting Li, Sylvine Raverdy, Jacopo F. Novelli, Panqing He, Natalie R. G. Dale, Amy M. Fife, Robert H. Barker, Martin L. Kramer, Wesley C. Van Voorhis, Clotilde K. S. Carlow, Ming-Wei Wang

**Affiliations:** 1 Division of Allergy & Infectious Diseases, University of Washington, Seattle, Washington, United States of America; 2 Genzyme Corporation, Waltham, Massachusetts, United States of America; 3 The National Center for Drug Screening, Shanghai Institute of Materia Medica, Chinese Academy of Sciences, Shanghai, China; 4 Division of Parasitology, New England Biolabs, Ipswich, Massachusetts, United States of America; Rush University Medical Center, United States of America

## Abstract

Cofactor-independent phosphoglycerate mutase (iPGAM) is essential for the growth of *C. elegans* but is absent from humans, suggesting its potential as a drug target in parasitic nematodes such as *Brugia malayi*, a cause of lymphatic filariasis (LF). iPGAM's active site is small and hydrophilic, implying that it may not be druggable, but another binding site might permit allosteric inhibition. As a comprehensive assessment of iPGAM's druggability, high-throughput screening (HTS) was conducted at two different locations: ∼220,000 compounds were tested against the *C. elegans* iPGAM by Genzyme Corporation, and ∼160,000 compounds were screened against the *B. malayi* iPGAM at the National Center for Drug Screening in Shanghai. iPGAM's catalytic activity was coupled to downstream glycolytic enzymes, resulting in NADH consumption, as monitored by a decline in visible-light absorbance at 340 nm. This assay performed well in both screens (Z′-factor >0.50) and identified two novel inhibitors that may be useful as chemical probes. However, these compounds have very modest potency against the *B. malayi* iPGAM (IC_50_ >10 µM) and represent isolated singleton hits rather than members of a common scaffold. Thus, despite the other appealing properties of the nematode iPGAMs, their low druggability makes them challenging to pursue as drug targets. This study illustrates a “druggability paradox” of target-based drug discovery: proteins are generally unsuitable for resource-intensive HTS unless they are considered druggable, yet druggability is often difficult to predict in the absence of HTS data.

## Author Summary

Parasitic worms like *Brugia malayi* cause widespread lymphatic filariasis (LF) in southeast Asia and sub-Saharan Africa. The adult worms causing most of the symptoms of LF are difficult to treat with existing drugs. As a possible step toward new LF drugs, we searched for inhibitors of the *B. malayi* cofactor-independent phosphoglycerate mutase (iPGAM), an enzyme thought to be critical to survival and development of this parasite. Despite testing over 100,000 compounds at each of two screening centers, we found only two compounds that consistently inhibited the *B. malayi* enzyme more strongly than the cofactor-dependent enzyme found in humans. These compounds have limited potency and are not especially great starting points for drug development. The 3-dimensional structure of iPGAM suggests that the active site is difficult to access from the surrounding solvent, which may partly explain our very low yield of inhibitors. We conclude that iPGAM may not be an ideal drug target in *B. malayi* or related organisms because it is difficult to inhibit with druglike compounds.

## Introduction

For a protein to advance as a potential drug target, it should not only be important in pathogen survival and/or virulence, but also must be “druggable,” *i.e.*, susceptible to modulation with drug-like compounds. Many otherwise promising proteins simply do not have binding pockets that lend themselves to therapeutic intervention [1], [2]. Historically, metabolic enzymes have been considered relatively druggable because (by definition) they bind small molecules, which can sometimes be mimicked by drugs [3]. Still, since enzymes' reactants may bear little resemblance to drugs in their hydrophilicity or other properties [4], a protein's amenability to therapeutic modulation cannot be guaranteed even if it is an enzyme. Another aspect of druggability concerns the chemical tractability of hit scaffolds identified through the HTS process. While assemblers of small molecule libraries strive to include tractable molecules, depending upon the source(s) of those libraries, not all scaffolds may be amenable to chemical modification.

Lymphatic filariasis (LF) is an infectious disease caused by the parasite nematodes *Wuchereria bancrofti*, *Brugia malayi*, and *Brugia timori*. Southeast Asia and sub-Saharan Africa harbor most of the world's ∼120 million current infections. It has been estimated that 40 million people suffer significant morbidity and/or disfigurement due to filariasis [5]. Most disfigurement is caused by adult-stage worms (macrofilariae), which are more impervious than immature worms (microfilariae) to current drugs such as diethylcarbamazine, ivermectin, and albendazole [6]. Thus, there is a strong need for new drugs, especially those that affect previously unexploited target proteins.

In the ongoing search for new LF drug targets, cofactor-independent phosphoglycerate mutase (iPGAM) has attracted significant interest. iPGAM catalyzes the interconversion of 2-phosphoglycerate (2-PG) and 3-phosphoglycerate (3-PG) in glycolysis and gluconeogenesis. The candidacy of iPGAM as a LF drug target is supported by several lines of evidence. RNAi knockdown of the *C. elegans* iPGAM, whose amino acid sequence is 70% identical to that of the *B. malayi* iPGAM, results in embryonic lethality or developmental defects (depending on the timing of the dsRNA injection), suggesting its functional importance in nematodes [7]. Selective inhibition of the parasite enzyme without harming the host should be possible, since mammals possess only a cofactor-dependent phosphoglycerate mutase (dPGAM), which differs greatly from iPGAM in structure, mechanism of action, and kinetic profile [8]. In particular, iPGAM is distinct from dPGAM in being catalytically active even in the absence of the cofactor 2,3-bisphosphoglycerate [9]. Finally, bacterially expressed iPGAMs from *B. malayi* and *C. elegans* have been purified and characterized [7], [8] and thus are readily available for high-throughput screening (HTS).

iPGAM's druggability – another key criterion in drug target prioritization, as noted above – has not yet been subjected to thorough experimental scrutiny, as far as we know. No potent inhibitors have been publicly reported to date, and the lack of a nematode iPGAM crystal structure further limits assessment of druggability. At the level of amino acid sequences, the closest iPGAMs with published structures are those from *Bacillus stearothermophilus* and *Bacillus anthracis* [10], [11], the former being 41% identical and 60% similar to the *B. malayi* iPGAM. The *Bacillus* structures show a monomeric protein with two domains: a phosphatase domain that removes the phosphate group from the glycerate substrate and a transferase domain that returns the phosphate to the substrate [10]. The two domains may twist to form open and closed conformations, with the open conformation apparently corresponding to an absence of substrate [11]. Thus, iPGAM's druggability could hinge partly on the fraction of time it spends in the open state, in which access to its active site is increased. However, this active site may not be especially druggable. The reactants (2-PG and 3-PG) are highly polar, and the nine amino acids that interact with them in the *Bacillus stearothermophilus* iPGAM (S62, H123, R153, D154, R185, R191, R261, R264, and K336) are all hydrophilic [10], [12]. Highly polar molecules (e.g., those with >5 hydrogen bond donors or >10 hydrogen bond acceptors [13]) are generally not “drug-like” in that they are poorly permeable through lipid membranes in the absence of a specific cellular transporter. The nine polar residues are all conserved in the *B. malayi* iPGAMiPGAM. Moreover, the iPGAM active site appears too small to accommodate additional, more hydrophobic moieties (Christophe Verlinde, personal communication). Thus, the active site of the *B. malayi* iPGAM is unlikely to be druggable in the sense of being bound by a sufficiently hydrophobic molecule.

The above analysis does not preclude the possibility of allosteric inhibition, however. In principle, allosteric inhibitors have the advantage of not needing to out-compete enzymes' substrates [14]. In practice, they have shown promise in studies of several infectious disease drug targets, including HIV reverse transcriptase [15], HIV integrase [16], hepatitis C virus NS5B polymerase [17], and *Bacillus anthracis* edema factor [18]. In the specific case of iPGAM, one can imagine an allosteric effector that forces the enzyme toward a closed conformation in the absence of substrate binding, thus preventing catalysis.

In the hope of finding *B. malayi* iPGAM inhibitors suitable for drug development, we performed high-throughput screens (HTS) of large compound collections at two sites: Genzyme Corporation (Waltham, MA, USA) and the National Center for Drug Screening (NCDS; Shanghai, China). In doing so, we took advantage of an innovative partnership (previously discussed in this journal [19]) between Novo Nordisk, the World Health Organization, and the NCDS. This partnership has enabled NCDS to screen a compound library formerly owned by Novo Nordisk, as exemplified by the present study and previous work [20].

## Methods

### General screening strategy

iPGAM is dependent on divalent cations [8] and, like all enzymes, is responsive to changes in substrate concentrations. We screened for iPGAM inhibitors under conditions of abundant Mg^2+^ and abundant substrate (3-PG), which approximate normal cellular conditions. In particular, partial inhibition of glycolysis may lead to a buildup of [3-PG], and we hoped to discover inhibitors (whether competitive or allosteric) that would be effective even in the face of elevated substrate levels. Likewise, we did not pre-incubate the enzyme with compounds before adding substrate because we wanted to identify compounds that could inhibit iPGAM under physiological conditions, i.e., with substrate present.

As in previous work [7], [8], conversion of 3-PG to 2-PG (*i.e.*, activity in the “forward”/glycolytic direction) was monitored via coupling of iPGAM to the downstream glycolytic enzymes enolase, pyruvate kinase (PyK), and lactate dehydrogenase (LDH). The primary assay readout was absorbance at 340 nm, reflecting NADH consumption by LDH, which in turn reflects upstream activity by PGAM, enolase, and PyK.

**Figure 1:**
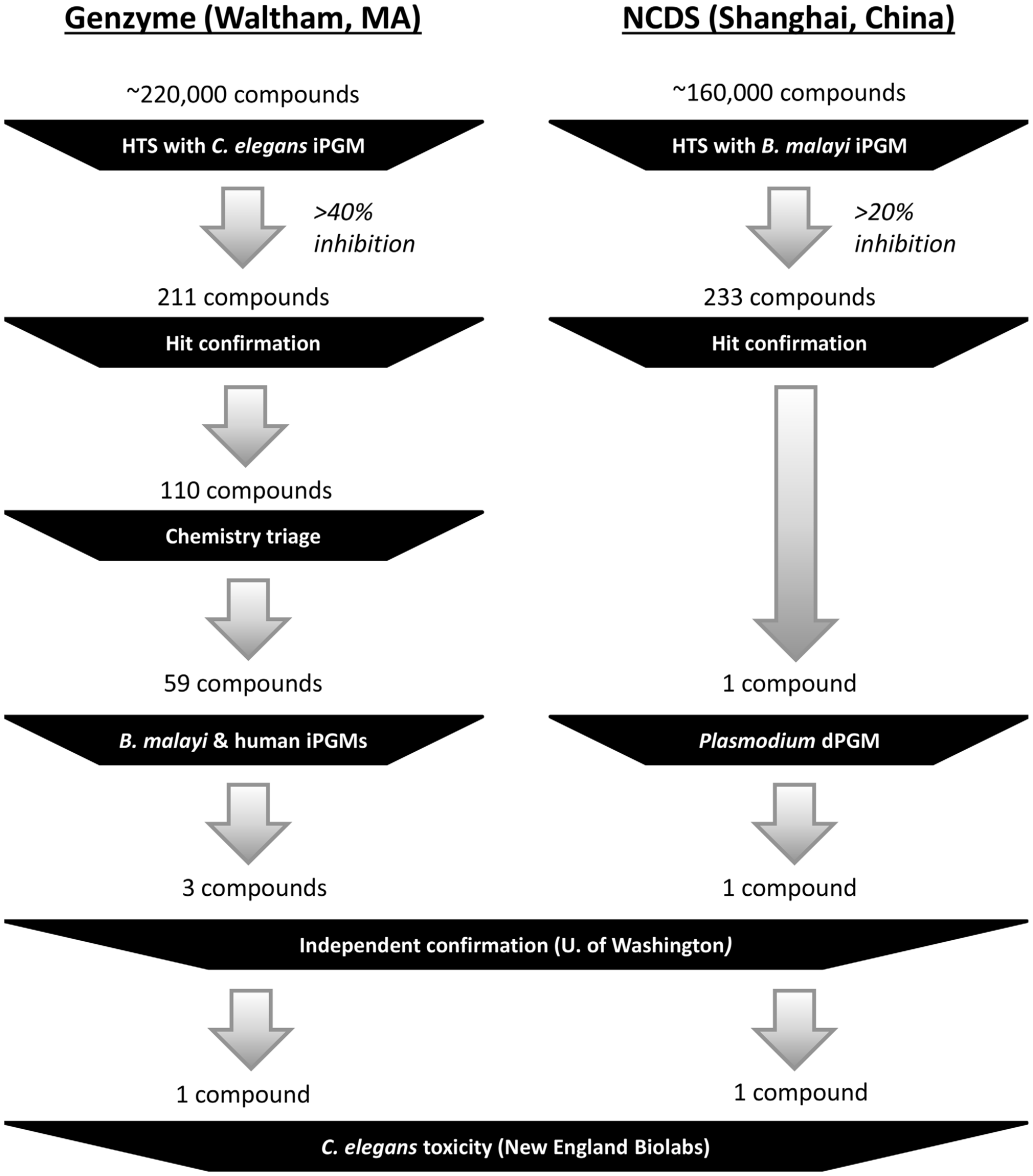
A summary of the screening workflow at the two HTS sites, along with the number of compounds passing through each step. See Methods for details.

The two screening centers' workflows were somewhat different (Fig. 1). At Genzyme, compounds giving >40% inhibition against the *C. elegans* iPGAM were cherry-picked and re-tested; reconfirmed hits were then analyzed for chemical tractability [21]; compounds deemed tractable were tested against the *B. malayi* iPGAM and human dPGAM; and compounds showing relatively potent and selective inhibition of *B. malayi* iPGAM (IC_50_ <30 µM and lower than the IC_50_ *vs*. human dPGAM) were advanced to independent confirmation in a luminescence-based assay and efficacy testing with *C. elegans* larvae. At NCDS, the HTS was followed by dose-response assays of compounds that gave >20% inhibition in the initial screen. The best NCDS hit was then tested for activity against the *P. falciparum* dPGAM (since the human dPGAM was not readily available at the time), confirmed independently in the luminescence-based assay, and tested for efficacy against *C. elegans* larvae.

Primary efficacy testing was done against *C. elegans* rather than *B. malayi* for reasons of convenience. Any compounds showing good potency versus *C. elegans* in vitro would have been advanced to a model of *B. malayi* infection in gerbils [22].

### Materials

Full-length, histidine-tagged iPGAM from *C. elegans* and *B. malayi* and full-length, histidine-tagged dPGAM from *Homo sapiens* and *Plasmodium falciparum* were expressed and purified as described previously [7], [8], [23]. 3-PG, adenosine diphosphate (ADP), nicotinamide adenine dinucleotide (NADH), PyK (from rabbit muscle, product P7768), LDH (from rabbit muscle, product L2500) and a PyK/LDH mixture (from rabbit muscle; product P0294) were purchased from Sigma-Aldrich (St. Louis, MO, USA). Enolase (from yeast; product #15515) was procured from Affymetrix (Santa Clara, CA, USA) and USB (Cleveland, OH, USA). Bovine serum albumin (BSA) was obtained from Amresco (Solon, OH, USA) and Sigma. 384-well plates came from BD Biosciences (Franklin Lakes, NJ, USA) and PerkinElmer (Waltham, MA, USA). Kinase-Glo was from Promega (Madison, WI, USA).

### Catalytic assay details

HTS assays were performed in 384-well plates (final volume: 50 µL per well). For the HTS at Genzyme, final assay concentrations were 30 mM Tris-HCl (pH 7); 5 mM MgSO_4_; 20 mM KCl; 1.5 mM 3-PG (3–4 times the K_m_; see Results below); 500 µM NADH; 3 mM ADP; 38.6 ng/mL *C. elegans* iPGAM; 2.5 Units/mL each of enolase, PyK, and LDH; 0.2% BSA and 10 µM of each compound tested (final DMSO concentration: 1%). For the HTS at NCDS, final concentrations were 30 mM Tris-HCl (pH 7.9); 5 mM MgSO_4_; 20 mM KCl; 3.5 mM 3-PG; 450 µM NADH; 2.5 mM ADP; 300 ng/mL *B. malayi* iPGAM; 2.265 U/mL enolase; 3.15 U/mL PyK; 4.71 U/mL LDH; 0.4 mg/mL BSA; and 5 µM of each compound tested (final DMSO concentration: 2%). At both locations the assay readout was absorbance at 340 nm. At Genzyme, absorbance data were taken as end-point readings on an Envision microplate reader (PerkinElmer) after 25 minutes of incubation at room temperature (∼20°C). At NCDS, data were collected kinetically (every 33 seconds) on a SpectraMax M2 microplate reader (Molecular Devices, Sunnyvale, CA, USA) during 15 minutes of incubation at room temperature following a 2-minute delay. HTS controls, representing the equivalent of enzyme inhibition, were wells with reduced [iPGAM] at Genzyme and wells with 50 µM or 200 µM tannic acid (discovered in preliminary Genzyme studies to reduce iPGAM activity) at NCDS.

### Independent confirmation of inhibition

To ensure robust, replicable results, hit compounds from the Genzyme and NCDS sites were shipped to the University of Washington for independent confirmation of inhibition of the *B. malayi* iPGAM using a separate batch of enzyme and a distinct assay readout. Catalytic assays were performed as described above except that ATP production by PyK was measured as luminescence at 528 nm following addition of Kinase-Glo, a luciferase-based reagent. A decrease in the slope of luminescence versus time was interpreted as inhibition of iPGAM.

### Compound libraries

Proprietary compound libraries housed at Genzyme and NCDS were screened. All compounds were pre-solubilized in 100% DMSO prior to use. The ∼220,000 compounds screened at Genzyme were taken from a library of ∼250,000 compounds from preferred vendors and internally synthesized compounds. This library emphasizes (A) drug-like or lead-like compounds, as judged by the criteria of compliance with the Rule of 5 [13] or Rule of 3 [24], compliance with Veber rules [25], and having similarity to known drugs according to fingerprint analysis; (B) heterocycles (∼2000 unique ring assemblies); and (C) natural product analogs (>7000). Over 40 screens have been conducted using this library, and most yielded usable chemical hits in other assays. The ∼160,000 compounds screened at NCDS were from a library of ∼325,000 synthetic compounds donated to NCDS by Novo Nordisk. The structural diversity of this library covers heterocycles, lactams, sulfonates, sulfonamides, amines, secondary amides, and natural product-derived compounds. Both libraries are intended to be relatively free of nonspecific aggregation-promoting compounds [26], but aggregators appearing as hits in the primary screen are generally filtered out during the hit confirmation process.

### *C. elegans* efficacy assay

L1 arrested larvae were resuspended in S Basal medium, with *E. coli* supplied as food and test compounds added from DMSO stocks to final concentrations of 0 to 75 µM. Worms were incubated in liquid culture in multi-well plates (100 µL per well) at 20°C for three days and then scored for growth defects/arrest. At least 16 wells (each containing 20 to 40 worms) were scored for each concentration of each compound. Only one generation of worms was followed.

### Accession numbers

The iPGAM from *B. malayi* has a GenBank ID of AY330617 and a UniProt ID of Q4VWF8. The iPGAM from *C. elegans* has a GenBank ID of AY594354 and a UniProt ID of G5EFZ1.

## Results/Discussion

**Figure 2:**
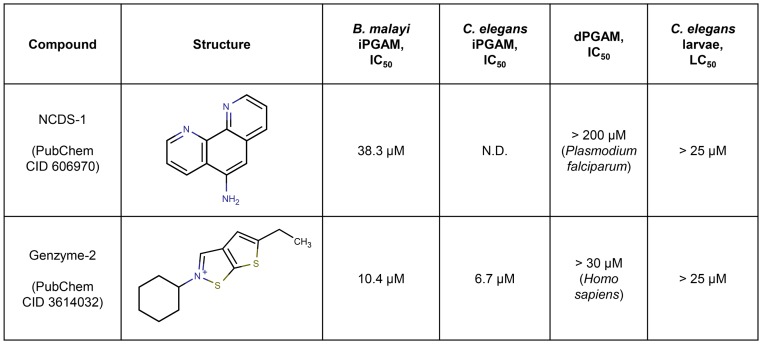
Novel inhibitors of *B. malayi* iPGAM. N.D. = Not determined.

**Figure 3:**
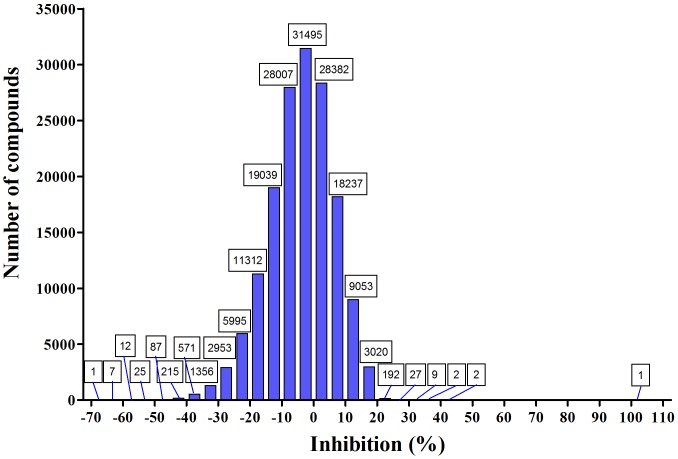
Results of NCDS's HTS of 160,000 compounds against the *B. malayi* iPGAM. The distribution of compounds is shown as a function of percent inhibition of the enzyme.

**Figure 4:**
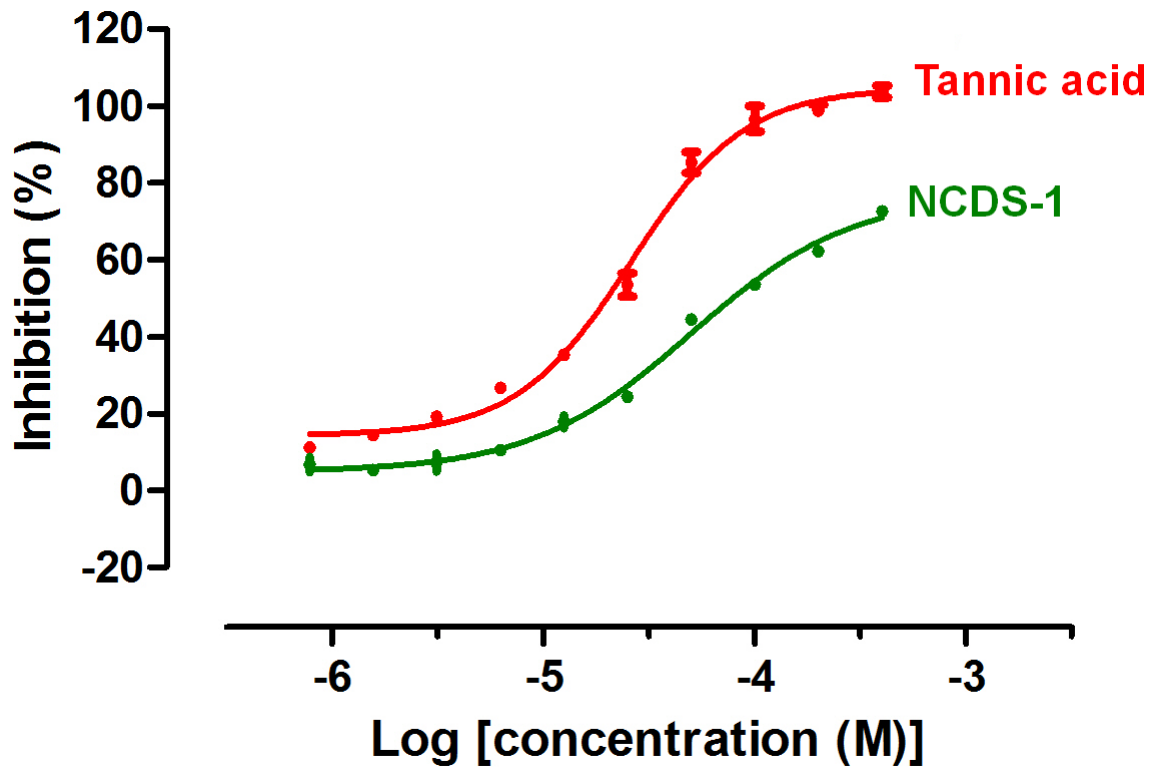
Dose-response curve of the confirmed hit NCDS-1. NCDS-1 and control inhibitor tannic acid were tested at concentrations from 0.78 µM to 400 µM. Note that tannic acid may inhibit iPGAM via nonspecific aggregation [40]; this would render it unsuitable as a chemical probe, but does not prevent its use as a control inhibitor for comparison of inhibited and uninhibited samples. Nonlinear regression and IC_50_ value were analyzed with GraphPad Prism software (GraphPad, San Diego, CA, USA). Data are expressed as means ± SEM of at least 3 independent experiments.

Our attempts to find specific inhibitors of the *B. malayi* iPGAM met with extremely limited success. The Genzyme screen tested ∼220,000 compounds at a concentration of 10 µM; it identified 110 confirmable hits against the *C. elegans* iPGAM, but only one of these compounds (Fig. 2) passed all of the follow-up steps shown in Fig. 1. The yield of the ∼160,000-compound screen at NCDS was equally low. 233 compounds (0.15%) initially appeared to show ≥20% inhibition of the *B. malayi* iPGAM at a concentration of 5 µM (Fig. 3), but of these, only one compound consistently gave an IC_50_ <50 µM (Fig. 4).

In theory, such an exceedingly low frequency of confirmable hits could reflect problems with (A) the performance of the assay, (B) the makeup and/or handling of the compound libraries, and/or (C) the enzymes being screened. We now address each of these possibilities.

**Figure 5:**
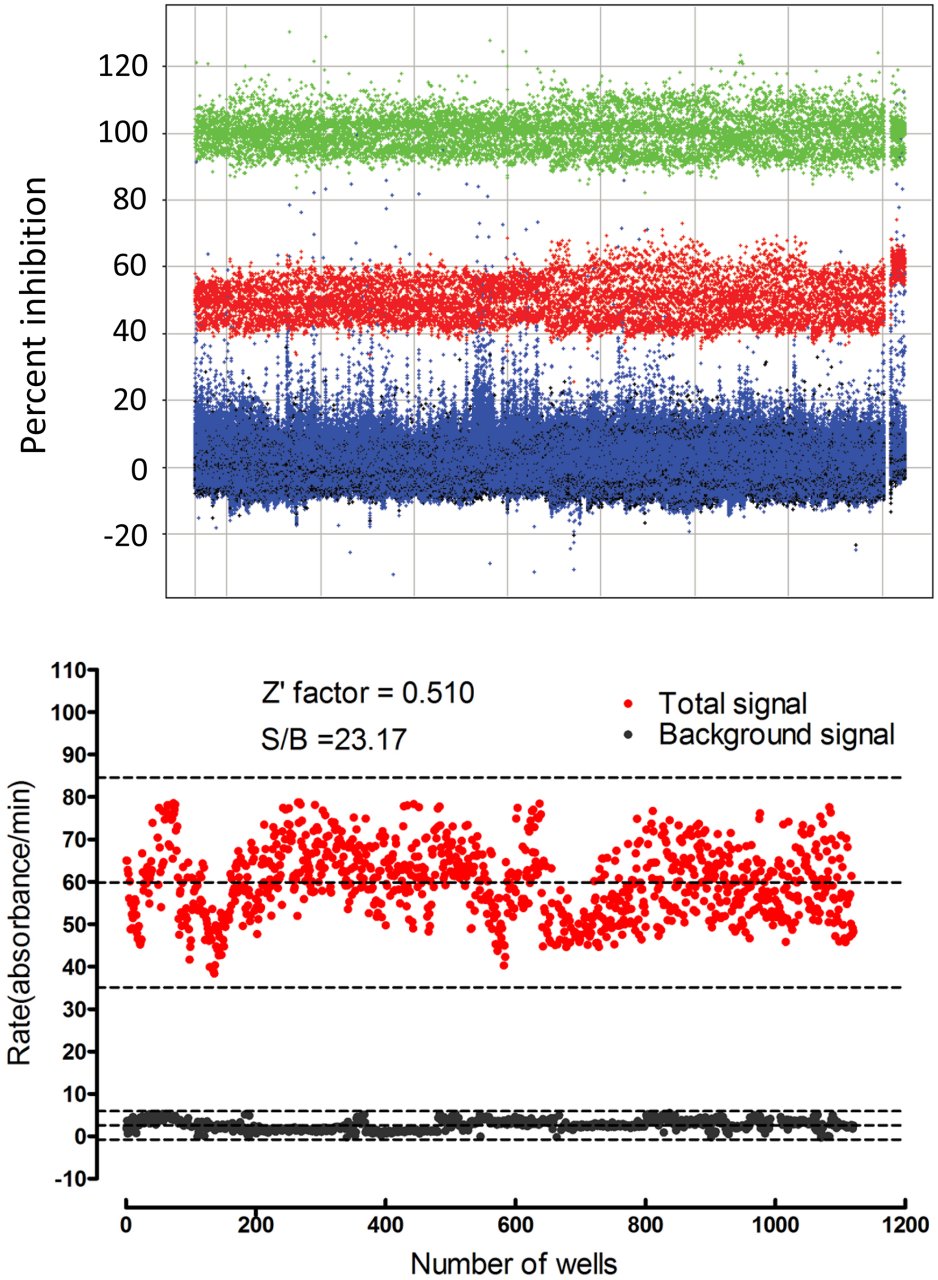
Robustness of the HTS assays performed at Genzyme (top) and NCDS (bottom). TOP: scattergram of percent inhibition data from all plates in the Genzyme HTS. Green: 100% inhibition control; red: 50% inhibition control; black: 0% inhibition control; blue: data for test compounds screened at 10 µM. BOTTOM: scattergram of NCDS data representing uninhibited iPGAM (2% DMSO, shown in red) and inhibited iPGAM (200 µM tannic acid, shown in black). 1120 wells for each condition, spread over seventy 384-well plates, are shown. Dashed lines indicate means ±3× standard deviation (SD).

Regarding assay performance (A), the standard measure of HTS quality is the Z′-factor, which reflects the means and variability of inhibited and uninhibited samples. Z′-factors range from 1 to below 0, with values above 0.5 indicating a robust assay [27]. Our mean Z′-factors were 0.65 for the Genzyme HTS and 0.51 for the NCDS HTS. Visual inspection of the HTS data from Genzyme and NCDS (Fig. 5) likewise showed large, clean separations between positive and negative controls. Therefore our assay seemed adequate for detecting iPGAM inhibitors.

Regarding compound libraries (B), there are two potential issues: the chemical “space” covered and compound stability. While library builders seek to ensure broad coverage of potentially drug-like chemical space, a lack of hits could reflect limits in this coverage. We also cannot be 100% certain that there were no problems with the storage, handling, or dispensing of the compounds tested. However, these same Genzyme/NCDS libraries have been used to identify potent hit compounds in screens against other target proteins (*e.g*., [20], [28]). Therefore it seems unlikely that the low hit rate was caused by systematic degradation or incorrect dispensing of compounds.

Finally, regarding the enzymes screened (C), enzymes were expressed in *E. coli* from plasmids originally generated by New England Biolabs. SDS-PAGE analysis gave the expected molecular masses of ∼57 kDa (*B. malayi*) and ∼59 kDa (*C. elegans*), as previously reported [7]. Newly determined K_m_'s for 3-PG of 0.37 mM (*B. malayi* iPGAM) and 0.38 mM (*C. elegans* iPGAM) were consistent with previously reported values [8] of 0.35 mM and 0.51 mM, respectively. Likewise, specific activities of the newly purified enzymes were similar to those reported previously (data not shown). Therefore, there is no indication that the enzyme stocks used in the HTS were misfolded or denatured, which would have hampered the search for inhibitors of the normal well-folded enzymes. However, it is notable that the Genzyme HTS used the *C. elegans* iPGAM rather than the *B. malayi* iPGAM, and that only a subset of the hits against the *C. elegans* iPGAM were subsequently tested against the *B. malayi* iPGAM. Given the strong amino-acid similarity (70% identity, 82% similarity) of the two enzymes [7], we would expect inhibitor profiles for these enzymes to be similar as well. Still, inhibitors specific for the *B. malayi* iPGAM might be missed in screening with the *C. elegans* iPGAM. The NCDS HTS directly addressed this possibility; it showed that using the *B. malayi* iPGAM in the primary screen did not appreciably increase the rate of hits against this enzyme.

Given these considerations, we believe that the low hit rates do not reflect any major experimental limitations, but instead may reflect iPGAM's poor druggability. There currently is no crystal structure for either the *B. malayi* or *C. elegans* enzymes; however, sequence homology [7] suggests that both of these are very similar to that of *Bacillus stearothermophilus*, for which the crystal structure has been resolved [12]. That structure suggests a peptide “gate” over the active site (confirmed in subsequent studies [29]) which may limit accessibility to potential inhibitors. Similar problems have been encountered for other enzyme targets. The crystal structure of cytosolic phospholipase A_2_ revealed that it contains an α-helical “lid” that can fold to cover the active site [30]. This feature has made it very difficult to obtain broad structural classes of inhibitors ([31] and John Leonard, personal communication).

The compounds shown in Fig. 2 may be useful as chemical probes in future studies of iPGAM, and thus represent an important outcome of our study. These compounds' potential application in drug development depends on several criteria, such as (A) potency against the target enzyme and target parasite, (B) chemical properties related to druglikeness and medicinal chemistry potential, (C) specificity of inhibition, and (D) activity of related compounds.

Regarding potency (A), neither hit compound had an IC_50_ *vs.* the *B. malayi* iPGAM of <10 µM, nor was either efficacious against *C. elegans* larvae at 25 µM. It has been proposed that, for anti-helminth hits to merit development into possible lead compounds, they should inhibit helminth motility by 50 to 100% at concentrations below 2 to 10 µg/mL [32], which would be 9 to 45 µM for a compound whose molecular weight is 223 (the average for the two compounds shown in Fig. 2). In this concentration range, our hits showed little or no activity against live worms. While factors like poor drug solubility, limited uptake into the worm gut, and limited transport across the cuticle could all affect activity in this assay, these compounds are not especially appealing as starting points for drug development.

Regarding chemical properties (B), both hits are reasonably drug-like and amenable to chemical synthesis and modification; e.g., they do not have limitations such as a high hydrophilicity or numerous chiral centers. Regarding off-target effects (C), PubChem BioAssay [33] shows that compound Genz-2 (PubChem CID 3614032) does not inhibit the other 9 targets against which it has been tested, while no bioassay data are available for NCDS-1 (PubChem 606970). Finally, of the five NCDS-screened compounds based on a scaffold of 1,10-phenanthroline, only the hit compound itself (5-amino-1,10-phenanthroline) gave any noticeable inhibition of the *B. malayi* iPGAM (D). This suggests that most changes to this compound's structure would eliminate its activity against iPGAM, and therefore that improvement of the hit compound's potency through medicinal chemistry would be difficult.

Thus, large-scale efforts at two different screening centers collectively failed to identify any high-priority compounds for drug development studies. In the absence of published empirical evidence that nematode iPGAMs can be potently and specifically modulated by drug-like molecules, advancing them as drug targets appears highly challenging and risky. We would also advise caution in the pursuit of non-nematode iPGAMs like the *Trypanosoma brucei* iPGAM [34], [35] as drug targets, since their druggability remains uncertain.

Our finding of poor nematode iPGAM druggability contrasts with a recent modeling study of the *Wuchereria bancrofti* iPGAM [36], which presents drug-like molecules proposed to be likely inhibitors. The authors developed a 3-D model of the *W. bancrofti* iPGAM, using the *B. stearothermophilus* iPGAM [12] as a template. They found that 63 residues were conserved among iPGAMs and that 53 residues contribute to binding pockets as defined by Q-SiteFinder [37]; the 19 residues belonging to both sets were termed the “common amino acid residues” (WB-iPGM_19cr_). When a virtual library of 2,344 small molecules were presented to the *W. bancrofti* iPGAM model in docking simulations, 65 were predicted to interact with at least one of the WB-iPGM_19cr_ residues, and 8 of these 65 (each linked to exactly one WB-iPGM_19cr_ residue) are considered to have good ADME/T properties and are “strongly recommended for further clinical trials.” We applaud this interest in the *W. bancrofti* iPGAM but note that none of the predicted inhibitors were tested experimentally.

Having invested considerable resources in screening nematode iPGAMs, only to find that they do not appear druggable, we must ask whether this disappointing outcome could have been predicted. The crystal structure of the *B. stearothermophilus* iPGAM does indicate a “gate” region peptide over the active site that could prevent access by potential inhibitors (see above [12], [29]). More recent studies on the crystalized structure of *Tryanosoma brucei* iPGAM also indicate that when crystalized in the presence of substrate, the substrate is buried and is not solvent-accessible, again suggesting a gate-like fold over the active site [35] that may increase the difficulty of finding inhibitors.

We also considered the possibility that allosteric inhibitors might be discovered as part of the screening process. *A priori* prediction of allosteric effects remains challenging; as summarized in one review, “In most cases, the novel allosteric binding sites could not have been predicted from the unliganded structure” [38]. When studying a protein without a solved crystal structure, such as the *B. malayi* iPGAM, the challenge increases further.

In conclusion, we suggest that target-based drug development suffers from a frustrating paradox: proteins are generally unsuitable for resource-intensive HTS unless they are considered druggable, yet druggability is often difficult to estimate in the absence of HTS data. Although improved druggability predictions [39] may eventually offer a way out of this paradox, a moderate level of risk currently appears unavoidable in the screening of many novel protein targets.
